# Vaccination coverage in Lebanon following the Syrian crisis: results from the district-based immunization coverage evaluation survey 2016

**DOI:** 10.1186/s12889-019-6418-9

**Published:** 2019-01-14

**Authors:** Ziad Mansour, Randa Hamadeh, Alissar Rady, M. Carolina Danovaro-Holliday, Kamal Fahmy, Racha Said, Lina Brandt, Ramy Warrak, Walid Ammar

**Affiliations:** 1 Connecting Research to Development, Beirut, Lebanon; 2 Ministry of Public Health, Beirut, Lebanon; 3 World Health Organization Lebanon Country Office, Beirut, Lebanon; 4 World Health Organization, Geneva, Switzerland; 5 World Health Organization Eastern Mediterranean Region Office, Cairo, Egypt

**Keywords:** Expanded Programme on immunization, Vaccination coverage, Coverage evaluation survey, Lebanon, Syrian refugees

## Abstract

**Background:**

Following the Syrian crisis, a substantial influx of Syrian refugees into Lebanon posed new challenges to optimal vaccination coverage for all children residing in the country. In 2016, the district-based immunization coverage evaluation survey (CES) assessed routine immunization coverage at the district level in Lebanon among children aged 12–59 months.

**Methods:**

A cross-sectional multistage cluster survey was conducted in all of Lebanon (with the exception of the Nabatieh district) using the World Health Organization (WHO) recommended Expanded Programme on Immunization (EPI) methodology adapted to the local context. A survey questionnaire consisting of closed and open-ended questions concerning demographic information and the child’s immunization status was administered to collect immunization status information.

**Results:**

Among surveyed children aged 12–59 months, irrespective of nationality, vaccination coverage at the national level for any recommended last dose was below the targeted 95%. Generally, vaccination coverage levels increased with age and were higher among Lebanese than Syrian children. However, large variations were revealed when coverage rates were analyzed at the district level. Vaccination was significantly associated with nationality, age, mother’s educational status and the place of vaccination. Common reasons for undervaccination included the child’s illness at the time of vaccine administration, vaccination fees, lack of awareness or a doctor’s advice not to vaccinate during campaigns.

**Conclusions:**

Substantial variability exists in vaccination coverage among children aged 12–59 months residing in different districts in Lebanon. Immunization coverage reached 90% or above only for the first doses of polio and pentavalent vaccines. A considerable dropout rate from the first dose of any vaccine is observed. Efforts to optimize coverage levels should include increased vaccination initiatives targeting both refugee children and children from vulnerable host communities, increased cooperation between public and private vaccine providers, improved training for vaccine providers to adhere to complete vaccine administration recommendations, and increased awareness among caregivers.

**Electronic supplementary material:**

The online version of this article (10.1186/s12889-019-6418-9) contains supplementary material, which is available to authorized users.

## Background

Established in 1987 in Lebanon, the Expanded Programme on Immunization (EPI) works toward inclusive vaccination coverage for all children [1]. Table 1 presents the national immunization schedule for children aged 0–59 months adopted by the Lebanese Ministry of Public Health (MoPH) in 2016 [2]. Routine immunization for children in Lebanon is provided by public and private entities, including more than 700 primary healthcare centers and dispensaries [3]. National coverage estimates suggest that generally more than 90% of children are being reached with mandatory vaccines [4]. Nevertheless, an outbreak of measles in 2013 and a strong increase in cases of mumps in 2015 indicate the vulnerability of the current Lebanese vaccination coverage system [5].

**Table 1:** Routine vaccination schedule for children aged 0–59 months in Lebanon [2]

Child’s age	Vaccine	Doses
at birth	HepB	Zero Dose
2 months	IPV	1st Dose
	Penta (DTP, Hib and HepB)	
4 months	OPV	2nd Dose
	Penta (DTP, Hib, HepB)	
6 months	OPV	3rd Dose
	Penta (DTP, Hib, HepB)	
9 months	Measles vaccine^a^	Zero Dose
12 months	MMR^a^	1st Dose
18 months	OPV	1st Booster
	Penta (DTP, Hib, HepB)	
	MMR^a^	2nd Dose
4–5 years	OPV	2nd Booster
	DTP	

HepB *Hepatitis B****,*** Hib *Haemophilus influenzae type b****,*** IPV *Inactivated Polio Vaccine****,*** DTP *Diphtheria, Tetanus, Pertussis****,*** OPV *Oral Polio Vaccine****,*** MMR *Measles, Mumps, Rubella*

^a^ *In this article, measles and MMR vaccines were entered as measles-containing vaccine (MCV) (either measles or MMR vaccines) and rubella-containing vaccine (RCV)*

The enormous influx of Syrian refugees into the country since 2011 has posed challenges to optimal provision of immunization services and access to quality immunization services [5]. Due to the disrupted provision of vaccines in Syria as well as the difficulty in accessing to healthcare services by refugees, many Syrian children lack optimal immunization coverage [6]. The Vulnerability Assessment of Syrian Refugees in Lebanon confirms incomplete vaccination coverage among 50% of all surveyed Syrian children [7]. Another vaccination coverage cluster survey in the North of Lebanon in 2015 identifies a decisively lower vaccination coverage level among Syrian children compared to children from host communities [8].

In this regard, the EPI program in Lebanon aims at “1) elevating routine vaccination coverage in every district to above 95%, 2) preserving Lebanon as polio-free […], and 3) eradicating measles and rubella by the end of year 2018” [1]. Scale-up routine immunization and vaccination campaigns are used to respond to the increased risk of disease outbreaks. Regular monitoring and coverage estimation permit the evaluation of these vaccination efforts. To assess routine immunization coverage at the district level in Lebanon, a coverage evaluation survey (CES) was conducted among children aged 12–59 months between December 2015 and June 2016 [9]. The resulting evidence helps provide a better understanding of the current status and determinants of immunization uptake in Lebanon and aims to inform relevant stakeholders about achievements and shortcomings of their attainments.

## Methods

A cross-sectional survey was conducted between December 2015 and June 2016 among caregivers of children aged 12–59 months in all of Lebanon except the district of Nabatieh and was designed to provide district-based vaccine coverage estimates [9].

### Sampling

The study sample included resident children in Lebanon, irrespective of their nationality. Sample size calculations were made at the district level, assuming a conservative vaccination coverage of 50%, a desired precision of ±5%, a probability of achieving that precision of 0.95 and a design effect of 2. This led to a required sample of 390 children per district, that is, a total sample of 10,140 children from 26 districts.

Following the World Health Organization (WHO) cluster evaluation survey methodology, 26 clusters were randomly selected in each district proportionally to the population estimates obtained from the Central Administration of Statistics, which are based on a population census of 2009, and the United Nations High Commissioner for Refugees [10–12]. The clusters that were likely to be sampled more than once were assigned a fixed number of starting points based on how often they would be selected with certainty. Fifteen children were recruited from each cluster, with each child being selected from a different household.

Households were identified by randomly selecting a landmark from a list of landmarks in each cluster identified prior to the study with the support of local authorities. From the selected landmark, a direction was chosen by spinning a pen or another sharp object to approach the first household. Subsequent households were visited according to proximity, selecting the nearest household to continue data collection. If the end of a street was reached, the neighboring street was chosen following a clockwise approach. Participants were recruited until the total number of 15 was reached within each cluster. In the event that more than one eligible child was found in the same household, each child’s name was written on a slip of paper and the participating child was chosen at random. If the parent or legal guardian was absent during the field visit, the household was revisited at least twice. The same applied to empty houses where fieldworkers were able to inquire from neighbors that an eligible child should be hosted. Facility traceback to find documented evidence of vaccination was piloted and was found not to be feasible.

### Data collection

Sixty data collectors worked on the ground to collect data throughout the districts. All fieldworkers underwent intensive training on field practices, interview techniques and ethical considerations, as well as pilot test activity. A survey questionnaire (Additional file 1) consisting of closed and open-ended questions about demographic information and the child’s immunization status was developed, reviewed and approved by the MoPH, the Lebanon country office of the WHO and the United Nations Children’s Fund (UNICEF). The tool was forward- and back-translated from English to Arabic in order to ensure consistency and pilot tested in the cadasters of Dekweneh and Nabaa in Lebanon. Data collection was performed using paper questionnaires and the electronic KoBoCollect application on tablets. Epidata software was used to enter any nonelectronically collected information. Pictures of the child’s (one or more) immunization card(s) available in the house were also taken. Further, when a child was known to have missed the vaccination, caregivers were asked about reasons why their child had not been vaccinated for each type of vaccine separately.

### Data analysis

A response rate of 94.3%, accounting for all surveyed cases irrespective of their nationality, led to a total of 9560 children; however, for this study, we excluded 245 (2.6%) children who were not Lebanese or Syrians living in the communities. Data were analyzed using Stata software, version 14. Descriptive analyses were presented as proportions and means with standard deviations where appropriate. National and district level vaccination coverage estimates and 95% confidence intervals (CI) were calculated based on the Taylor Series Linearization method to retrieve results for the entire sample. National estimates took into account the sampling design (stratum, district and governorate-specific weight). Vaccination cards were used for validating received vaccinations, and coverage rates of children with a well-documented vaccination card were presented. If the vaccination card was incomplete or missing, the recall of caregivers was considered to assess the child’s vaccination status. Dropout rates were calculated as the difference in coverage between the first and third doses for each of the following: polio, DTP, HepB and Hib; the first and second doses for MCV; and the first dose of DTP and first dose of MCV. A multivariable logistic regression analysis of completed vaccination coverage, also accounting for the sampling design, was performed for each vaccine separately. Significance was considered at a *p*-value < 0.05 following a t-distribution.

### Ethical considerations

Before each interview, oral informed consent was obtained from the child’s caretaker. Written consent was not obtained as this is not a common practice for this type of studies in Lebanon, given the low levels of literacy among certain populations and the non-sensitive nature of the information obtained. All participants were informed of their completely free choice of participation and the strict application of confidentiality to any of the participants’ disclosed information. Prior to the start of any interview, participants received a brief but thorough explanation of the scope and aim of the survey. The Institutional Review Board at Sagesse University approved the study. Only the study team handled the database and pictures to ensure data confidentiality.

## Results

### Survey population

In total, 9315 Lebanese and Syrian children participated in the survey (Table 2). Included households had a mean size of 5.17 individuals (+SD 2.24) and a mean number of eligible children of 1.59 (+SD 0.88). Among the surveyed children, 7136 (76.6%) were Lebanese, and 2179 (23.4%) were Syrian. The sample was almost equally distributed among males and females and among the four age groups. Vaccination cards were presented for 5713 (61.4%) children, whereas for 3375 (36.2%) children, vaccination status was assessed through the recall of caregivers. Additionally, 227 children (2.4%) in the sample never received any vaccination. Enrolled children predominantly lived either in a rented (47.9%) or an owned (48.8%) house. Furthermore, 8559 (91.9%) of the caregivers were married with a mean age of 30.9 years (+SD 6.7). In addition, 5336 of fathers (57.2%) and 5089 (54.6%) of mothers completed at least their secondary level of education. Among fathers, approximately two-thirds (66.3%) had a full-time job compared to one-third of mothers (30.2%). Table 3 presents key sociodemographic factors by nationality.

**Table 2:** Household distribution by governorate/district (unweighted), Lebanon CES 2016

Governorate District					Household characteristics			
	Total		Lebanese		Syrian		Household size	Number of eligible children aged 12–59 months
	Number	Percentage	Number	Percentage	Number	Percentage	Mean + SD	Mean + SD
Akkar								
Akkar	390	4.2	320	4.5	70	3.2	5.5 + 2.7	2.0 + 1.3
Baalbek-Hermel								
Baalbek	390	4.2	262	3.7	128	5.9	5.5 + 2.3	1.6 + 0.6
Hermel	390	4.2	297	4.2	93	4.3	5.2 + 2.2	1.7 + 1.0
Beirut								
Beirut	380	4.1	317	4.4	63	2.9	4.7 + 2.1	1.3 + 0.5
Bekaa								
Rashaya	388	4.2	319	4.5	69	3.2	5.1 + 1.7	1.3 + 0.5
West Bekaa	383	4.1	285	4.0	98	4.5	5.4 + 2.3	1.4 + 0.7
Zahle	231	2.5	174	2.4	57	2.6	5.4 + 1.9	1.7 + 0.9
Mount Lebanon								
Aley	385	4.1	320	4.5	65	3.0	4.6 + 1.9	1.4 + 0.7
Baabda	380	4.1	276	3.9	104	4.8	4.8 + 1.7	1.3 + 0.6
Chouf	377	4.1	321	4.5	56	2.6	5.3 + 2.7	1.6 + 1.0
El-Metn	364	3.9	277	3.9	87	4.0	5.5 + 2.5	1.5 + 0.7
Jbeil	388	4.2	304	4.3	84	3.9	4.8 + 2.1	1.6 + 0.7
Keserwan	375	4.0	294	4.1	81	3.7	4.6 + 2.1	1.4 + 0.6
Nabatieh								
Bint Jbeil	390	4.2	286	4.0	104	4.8	4.5 + 1.6	1.7 + 0.9
Hasbaya	381	4.1	267	3.7	114	5.2	5.4 + 2.2	2.3 + 1.4
Marjeyoun	388	4.1	299	4.2	89	4.1	4.3 + 1.7	1.9 + 1.1
Nabatieh^a^	–	–	–	–	–	–	–	–
North								
Batroun	381	4.1	277	3.9	104	4.8	5.3 + 2.0	1.4 + 0.6
Bcharre	384	4.1	281	3.9	103	4.7	4.8 + 1.5	1.9 + 0.9
Koura	390	4.2	309	4.3	81	3.7	4.9 + 1.9	1.5 + 0.7
Minieh-Donieh	390	4.2	287	4.0	103	4.7	5.7 + 2.4	1.6 + 0.8
Tripoli	383	4.1	308	4.3	75	3.4	5.7 + 2.6	1.7 + 1.3
Zgharta	390	4.2	353	5.0	37	1.7	4.1 + 1.1	1.2 + 0.4
South								
Jezzine	368	4.0	251	3.5	117	5.4	6.0 + 2.1	1.7 + 0.8
Saida	322	3.5	234	3.3	88	4.0	6.4 + 3.1	1.6 + 0.8
Sour	327	3.5	218	3.1	109	5.0	5.8 + 3.3	1.1 + 0.8
Total	9315	100.0	7136	76.6	2179	23.4	5.2 + 2.2	1.6 + 0.9

^a^Nabatieh was not included

**Table 3:** Key sociodemographic factors (unweighted), Lebanon CES 2016

Characteristics	Total (*n* = 9315)		Lebanese (*n* = 7136)		Syrian (*n* = 2179)	
	Number	Percentage	Number	Percentage	Number	Percentage
Sex of the child						
Male	5044	54.1	3851	54.0	1193	54.7
Female	4271	45.9	3285	46.0	986	45.3
Age of the child						
12–23 months	2579	27.7	1928	27.0	651	29.9
24–35 months	2265	24.3	1747	24.5	518	23.8
36–47 months	1920	20.6	1462	20.5	458	21.0
48–59 months	2551	27.4	1999	28.0	552	25.3
Place of residency of the caregiver						
Rented house / apartment	4461	47.9	2562	35.9	1899	87.2
Owned house / apartment	4547	48.8	4420	61.9	127	5.8
Informal settlement	130	1.5	40	0.6	90	4.1
Collective shelter	78	0.8	37	0.5	41	1.9
Other	49	0.5	34	0.5	15	0.7
Refused to answer	50	0.5	43	0.6	7	0.3
Social status of the caregiver						
Single	451	4.9	327	4.6	124	5.7
Married	8559	91.9	6567	92.0	1992	91.3
Divorced	137	1.5	103	1.4	34	1.6
Widowed man	51	0.5	45	0.6	6	0.3
Widowed woman	88	0.9	69	1.0	19	0.9
Refused to answer	29	0.3	25	0.4	4	0.2
Age of the caregiver	30.9 + 6.7		31.2 + 6.7		29.7 + 6.7	
Median	30.0		30.3		28.9	
Father’s educational status						
Doesn’t know how to read and write	605	6.5	312	4.4	293	13.5
Knows how to read and write	1412	15.2	795	11.1	617	28.3
Primary/Complementary level	1830	19.7	1319	18.5	511	23.5
Secondary level	1774	19.0	1609	22.6	165	7.6
Post school technical level	1035	11.1	936	13.1	99	4.5
University level	2527	27.1	2054	28.8	473	21.7
Doesn’t know/Doesn’t remember	39	0.4	33	0.5	6	0.3
Refused to answer	93	1.0	78	1.0	15	0.6
Mother’s educational status						
Doesn’t know how to read and write	695	7.5	312	4.4	383	17.6
Knows how to read and write	1099	11.8	626	8.8	473	21.7
Primary/Complementary level	2330	25.0	1550	21.7	780	35.8
Secondary level	1856	19.9	1614	22.6	242	11.1
Post school technical level	1243	13.3	1033	14.5	210	9.6
University level	1990	21.4	1915	26.8	75	3.4
Doesn’t know/Doesn’t remember	20	0.2	14	0.2	6	0.3
Refused to answer	82	0.9	72	1.0	10	0.5
Father’s employment status						
Full-time employee	6177	66.3	5127	71.8	1050	48.2
Part-time employee	2204	23.7	1462	20.5	742	34.1
Unemployed	705	7.6	348	4.9	357	16.4
Retiree	48	0.5	43	0.6	5	0.2
Refused to answer	181	1.9	156	2.2	25	1.1
Mother’s employment status						
Full-time employee	2816	30.2	2344	32.8	472	21.7
Part-time employee	1772	19.0	1375	19.3	397	18.2
Unemployed	4595	49.4	3302	46.3	1293	59.3
Retiree	21	0.2	14	0.2	7	0.3
Refused to answer	111	1.2	101	1.4	10	0.5

### Routine vaccination coverage

Based on vaccination cards and parental recall, vaccination coverage among children aged 12–59 months was below the targeted 95%, except for the first doses of pentavalent and polio among Lebanese children (Table 4). Complete vaccination coverage of each vaccine separately revealed the highest coverage for the third dose of Hib (88.1% [95% CI: 87.0–89.2] among Lebanese, 78.7% [95% CI: 76.2–81.0] among Syrians) and the lowest coverage for the second dose of MCV (64.2% [95% CI: 62.4–66.0] among Lebanese, 50.1% [95% CI: 47.1–53.1] among Syrians). Dropout rates ranged from 6.7% (Hib 1 – Hib 3) to 18.9% (MCV 1 – MCV 2) among Lebanese and 10.2% (Hib 1 – Hib 3) to 23.4% (MCV 1 – MCV 2) among Syrian children.

**Table 4:** Routine vaccination coverage according to cards or card information and caregivers’ recall, Lebanon CES 2016

Vaccine	Lebanese (n = 7136)		Syrian (n = 2179)	
Source	Card (*n* = 4363)	Card+Recall^a^ (n = 7136)	Card (*n* = 1350)	Card+Recall^a^ (n = 2179)
	Percentage^b^ [95% CI]	Percentage [95% CI]	Percentage^b^ [95% CI]	Percentage [95% CI]
HepB 0 dose	86.6 [84.8–88.2]	85.5 [84.0–86.8]	67.0 [63.0–70.8]	70.7 [67.6–73.6]
Polio 1st dose	96.1 [95.2–96.8]	95.4 [94.6–96.0]	94.5 [92.2–96.2]	92.3 [90.2–94.0]
Polio 2nd dose	91.5 [90.3–92.6]	91.9 [90.9–92.8]	84.5 [81.3–87.2]	84.7 [82.1–87.0]
Polio 3rd dose	87.4 [86.0–88.7]	87.2 [86.0–88.4]	76.5 [73.1–79.6]	76.4 [73.6–79.0]
Dropout (Polio 1-Polio 3)	8.7	8.2	18.0	15.9
DTP 1st dose	95.9 [95.0–96.6]	95.9 [95.1–96.5]	89.4 [86.7–91.6]	89.9 [87.7–91.7]
DTP 2nd dose	92.5 [91.4–93.4]	90.1 [89.0–91.1]	83.7 [80.8–86.2]	82.0 [79.7–84.1]
DTP 3rd dose	88.8 [87.4–90.0]	87.7 [86.5–88.9]	76.6 [73.5–79.5]	77.5 [75.1–79.8]
Dropout (DTP 1-DTP 3)	7.1	8.2	12.8	12.4
HepB 1st dose	95.2 [94.2–96.0]	94.9 [94.2–95.6]	87.9 [85.1–90.2]	89.0 [86.8–90.9]
HepB 2nd dose	91.9 [90.7–92.9]	89.6 [88.5–90.7]	82.2 [79.1–84.9]	82.8 [80.4–85.0]
HepB 3rd dose	84.8 [83.1–86.2]	85.1 [83.7–86.3]	71.5 [68.0–74.8]	76.1 [73.3–78.6]
Dropout (HepB 1-HepB 3)	10.4	9.8	16.4	12.9
Hib 1st dose	95.3 [94.3–96.2]	94.8 [94.1–95.5]	88.4 [85.6–90.7]	88.9 [86.8–90.8]
Hib 2nd dose	92.3 [91.2–93.3]	90.5 [89.4–91.4]	83.4 [80.5–86.1]	83.5 [81.1–85.6]
Hib 3rd dose	88.7 [87.3–89.9]	88.1 [87.0–89.2]	76.0 [72.7–79.0]	78.7 [76.2–81.0]
Dropout (Hib 1-Hib 3)	6.6	6.7	12.4	10.2
MCV 1st dose	86.7 [85.3–88.0]	83.1 [81.7–84.4]	79.3 [76.1–82.1]	73.5 [70.6–76.3]
MCV 2nd dose	64.8 [62.7–66.9]	64.2 [62.4–66.0]	51.6 [48.1–55.1]	50.1 [47.1–53.1]
Dropout (MCV 1-MCV 2)	21.9	18.9	27.7	23.4
Dropout (DTP 1-MCV 1)	9.2	12.8	10.1	16.4
RCV 1st dose	72.4 [70.3–74.4]	70.8 [69.0–72.6]	69.2 [65.4–72.7]	62.1 [59.0–65.0]

^a^If the vaccination card was missing, the recall of caregivers was considered to assess the child’s vaccination status

^b^The denominator is the number of children with a well-documented vaccination card

### Routine vaccination coverage by district

**Fig. 1:**
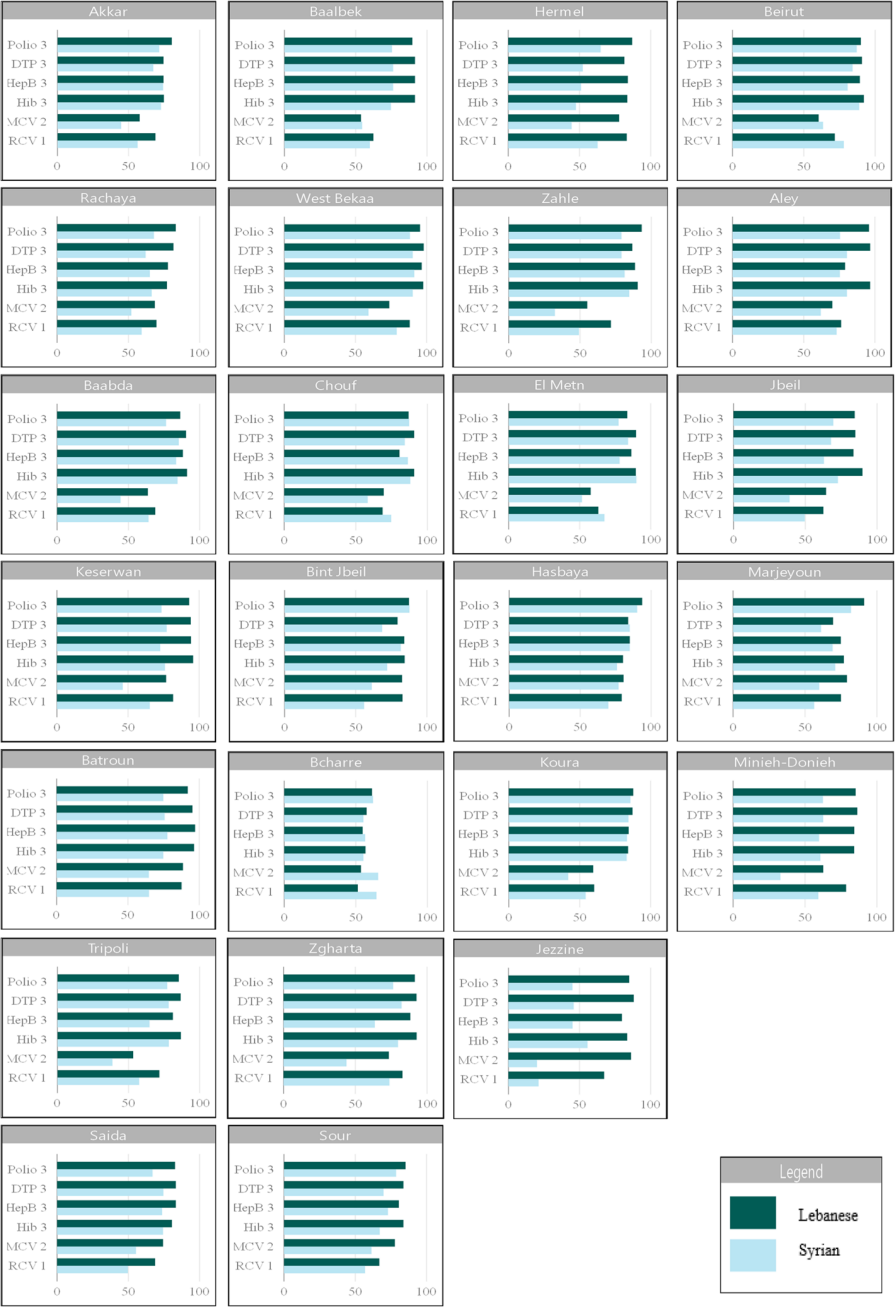
Routine vaccination coverage at the district level among children aged 12–59 months, Lebanon CES 2016

The coverage of any routinely administered vaccine differed between districts in Lebanon (Fig. 1). The difference in coverage among children aged 12–59 months for the third dose of polio ranged from 61.3% [95% CI: 50.9–70.7] in Bcharre to 95.9% [95% CI: 93.4–97.4] in Aley among Lebanese and from 45.0% [95% CI: 21.6–47.5] in Jezzine to 90.2% [95% CI: 81.5–95.1] in Hasbaya among Syrians. Similar variations were identified for all doses and all types of vaccines. However, the majority of districts presented an immunization level at the upper end of the targeted coverage. It was noted that more than 90.0% coverage of the first dose of polio was observed in all districts for Lebanese children, with the exception of Bcharre (78.9% [95% CI: 67.1–87.3]). Similarly, Syrians had a coverage of more than 90.0% in most districts for polio dose one; however, the vaccination coverage was lowest in Sour (87.2% [95% CI: 79.7–92.1]), Batroun (83.3% [95% CI: 60.1–94.3]), Jezzine (77.0% [95% CI: 63.2–86.6]), Rachaya (76.6% [95% CI: 63.8–85.9]), and Saida (72.7% [95% CI: 55.9–84.9]). The earlier ascertained disparity in coverage between Lebanese and Syrians changed when rates were analyzed at the district level. While overall coverage was generally higher among Lebanese than Syrians in Lebanon, large variations were revealed when focusing on various geographical areas.

### Factors associated with completed vaccination

As shown in Table 5, nationality was significantly associated with all types of vaccines except RCV, with Lebanese having significantly higher odds for completing vaccine coverage compared to Syrians, adjusting for other variables in the analysis. The child’s sex was not a significant predictor of vaccination coverage. The odds of having completed vaccination for all routine vaccinations increased with age. Children who received vaccinations at a private clinic were more likely to reach complete coverage for all types of vaccines, with the exception of polio and MCV. Mothers’ educational status significantly increased the odds for a child to be vaccinated, with mothers who completed their primary level of education or higher having higher odds of having a vaccinated child compared to illiterate mothers. This association was significant for all types of vaccines excluding HepB.

**Table 5:** Crude and adjusted odds ratios for sociodemographic factors associated with full vaccination, Lebanon CES 2016

Sociodemographic factors	Type of Vaccine											
	Polio 3		DTP 3		HepB 3		Hib 3		MCV 2		RCV 1	
Odds Ratio^a^	Crude [95% CI]	Adjusted [95% CI]	Crude [95% CI]	Adjusted [95% CI]	Crude [95% CI]	Adjusted [95% CI]	Crude [95% CI]	Adjusted [95% CI]	Crude [95% CI]	Adjusted [95% CI]	Crude [95% CI]	Adjusted [95% CI]
Nationality												
Lebanese[ref]												
Syrian	0.5* [0.4–0.6]	0.6* [0.5–0.7]	0.5* [0.4–0.6]	0.6* [0.5–0.8]	0.6* [0.5–0.7]	0.7* [0.5–0.8]	0.5* [0.4–0.6]	0.7* [0.6–0.8]	0.6* [0.5–0.6]	0.7* [0.6–0.8]	0.7* [0.6–0.8]	0.9 [0.7–1.0]
Sex												
Male[ref]												
Female	0.9 [0.8–1.1]	0.9 [0.8–1.1]	0.9 [0.8–1.1]	1.0 [0.8–1.2]	0.9 [0.8–1.1]	0.9 [0.8–1.1]	1.0 [0.8–1.1]	1.0 [0.9–1.2]	1.0 [0.9–1.1]	1.0 [0.9–1.1]	1.0 [0.9–1.1]	1.0 [0.9–1.1]
Age												
12–23 months[ref]												
24–35 months	1.2 [1.0–1.5]	1.3* [1.1–1.6]	1.1 [0.9–1.4]	1.2 [0.9–1.5]	0.9 [0.8–1.2]	1.0 [0.8–1.2]	1.1 [0.9–1.3]	1.1 [0.9–1.4]	1.8* [1.6–2.2]	1.9* [1.6–2.2]	1.5* [1.2–1.7]	1.5* [1.3–1.8]
36–47 months	1.2 [0.9–1.5]	1.3* [1.1–1.7]	1.2 [0.9–1.5]	1.3* [1.1–1.7]	1.0 [0.8–1.3]	1.1 [0.9–1.4]	1.1 [0.9–1.4]	1.2 [0.9–1.5]	1.9* [1.6–2.2]	2.1* [1.8–2.5]	1.9* [1.6–2.2]	2.0* [1.7–2.4]
48–59 months	1.4* [1.1–1.8]	1.7* [1.3–2.2]	1.2 [1.0–1.5]	1.5* [1.2–1.9]	1.2 [0.9–1.4]	1.3* [1.1–1.7]	1.1 [0.9–1.4]	1.4* [1.1–1.7]	2.5* [2.1–2.9]	2.9* [2.5–3.5]	2.4* [2.0–2.9]	2.8* [2.3–3.4]
Place of last vaccination												
Health facility[ref]												
Private clinic	1.4* [1.2–1.7]	1.1 [0.9–1.3]	1.7* [1.4–2.1]	1.3* [1.1–1.6]	1.5* [1.3–1.8]	1.3* [1.1–1.5]	1.9* [1.5–2.3]	1.4* [1.1–1.8]	1.4* [1.2–1.6]	1.1 [0.9–1.3]	1.5* [1.3–1.7]	1.3* [1.1–1.5]
Other	0.7* [0.5–0.9]	0.7* [0.5–0.9]	0.7* [0.5–0.9]	0.7* [0.5–0.9]	0.8* [0.6–0.9]	0.7* [0.5–0.9]	0.8 [0.6–1.1]	0.8 [0.6–1.1]	0.5* [0.4–0.7]	0.5* [0.4–0.6]	0.6* [0.5–0.7]	0.5* [0.4–0.7]
Mothers’ educational status												
Doesn’t know how to read and write[ref]												
Knows how to read and write	1.4* [1.1–1.9]	1.2 [0.9–1.7]	1.2 [0.9–1.6]	1.0 [0.7–1.4]	1.2 [0.9–1.7]	1.0 [0.7–1.4]	1.4* [1.1–1.8]	1.2 [0.8–1.6]	1.2 [0.9–1.7]	1.2 [0.9–1.6]	1.4* [1.1–1.8]	1.3 [1.0–1.7]
Primary/complementary level	1.9* [1.4–2.6]	1.4* [1.1–1.9]	1.5* [1.1–2.0]	1.0 [0.7–1.4]	1.4* [1.1–2.0]	1.0 [0.7–1.4]	1.7* [1.3–2.3]	1.2 [0.9–1.7]	1.4* [1.1–1.7]	1.2 [0.9–1.5]	1.8* [1.4–2.3]	1.5* [1.2–2.0]
Secondary level	2.7* [2.0–3.7]	1.7* [1.2–2.4]	2.0* [1.5–2.7]	1.1 [0.8–1.6]	1.8* [1.3–2.5]	1.1 [0.8–1.6]	2.5* [1.8–3.5]	1.5* [1.1–2.2]	2.3* [1.7–3.0]	1.7* [1.3–2.4]	2.0* [1.5–2.6]	1.6* [1.2–2.1]
Post-school technical level	1.9* [1.3–2.6]	1.3 [0.9–2.0]	1.9* [1.3–2.7]	1.2 [0.8–1.9]	1.6* [1.1–2.3]	1.0 [0.7–1.6]	1.6* [1.2–2.3]	1.1 [0.8–1.6]	1.8* [1.4–2.3]	1.5* [1.1–1.9]	2.3* [1.7–3.1]	1.9* [1.4–2.6]
University level	2.9* [2.1–4.0]	2.0* [1.4–3.0]	2.9* [2.1–4.1]	1.8* [1.2–2.8]	1.8* [1.3–2.5]	1.1 [0.8–1.6]	3.3* [2.4–4.5]	2.2* [1.5–3.1]	2.3* [1.8–3.0]	1.9* [1.4–2.5]	2.1* [1.7–2.8]	1.7* [1.3–2.2]

*P-values are significant at α = 0.05 level of significance. P-values were based on a t-distribution

^a^Crude odds ratios were based on a weighted simple logistic regression of completed vaccination coverage. Adjusted odds ratios were based on a weighted multivariable logistic regression model of completed vaccination coverage accounting for all the variables in the model. Both analyses took into consideration the sampling design and were performed for each vaccine separately

### Reasons for Undervaccination

The child being sick at the time for administering the vaccine was one of the main justifications for undervaccination of any kind of vaccine (3.1% for Hib up to 33.3% for MCV). Other common explanations were the inability to pay the fees for vaccination (4.7% for IPV up to 17.5% for Hib), lack of awareness of the need for immunization (4.4% for MCV up to 15.5% for OPV) or the vaccine’s importance (2.1% for RCV up to 6.4% for OPV). In many other cases, caregivers stated that the doctor did not advise vaccinating the child, which was most commonly reported for IPV (17.5%) and HepB (10.8%). Administration of Hib was often denied because parents did not trust the quality of the vaccine (18.4%). Other common reasons for undervaccination were the lack of availability of the vaccine, the child’s age or the reluctance of the parents to vaccinate their child. These reasons were not significantly different for Lebanese versus Syrian children for all types of vaccines.

## Discussion

Despite tremendous efforts to optimize immunization coverage, major challenges to reaching every child with needed protection against preventable diseases in Lebanon persist. National estimates of vaccine coverage do not detect gaps that are only revealed when looking at the district level. This study highlights substantial variability in vaccination coverage among children aged 12–59 months residing in different districts. While some areas present coverage rates up to almost 100%, others indicate inadequacies in the provision of vaccination services with coverage rates as low as 20.1%. According to the routine vaccination schedule of the MoPH, at the age of 12 months, a child should have received at least three doses of polio vaccine, DTP, and Hib, four doses of HepB vaccine and at least one dose of MCV [2]. The findings show, however, that routine vaccination coverage falls below levels that provide sufficient population immunity to prevent outbreaks [13]. Immunization coverage reaches 95% or above only for the first doses of pentavalent and polio among Lebanese children. Moreover, dropout rates higher than the 10.0% cutoff point set by the WHO were noted for all vaccines administered to Syrian children and for MCV among Lebanese children [10]. These dropouts are indicative of delayed follow-up or missed opportunities to administer vaccines on time.

Insufficient or missing vaccination coverage may result from multiple causes, including immunization system weaknesses, children’s family characteristics and parental attitudes or knowledge [14, 15]. In line with previously conducted research in Lebanon, this study shows discrepancies in the vaccination coverage for Lebanese and Syrian children [8]. These discrepancies, with large variations at the district level, suggest gaps in targeting vulnerable children. A series of vaccination campaigns and other vaccination initiatives in 2014, 2015 and 2016 following the influx of Syrians into Lebanon was established, which achieved adequate coverage levels for polio and measles [1]. Nevertheless, the study results highlight the importance of continuing vaccination efforts and extending initiatives to those children left behind in previous campaigns, particularly refugee and vulnerable children living in host communities. The additional challenges to the Lebanese healthcare system in recent years have stretched services and public infrastructure to the maximum to cope with the unprecedented increase in demand for vaccines [16].

In addition to the discrepancy in vaccination coverage associated with the child’s nationality, the results from this study suggest a difference in vaccine administration compliance between public and private providers. In Lebanon, children can receive vaccinations at public or private facilities [1]. The vaccine market is well established, and different types of vaccines are available. However, this privilege is mostly reserved for those receiving their vaccinations at private clinics, which administer types of vaccines that are often different from those provided by public healthcare facilities due to contractual and financial circumstances. The diverse forms of vaccinations received create difficulties in tracking where a child received the vaccination and create obstacles to assessing vaccination coverage and its completeness. Good record keeping for vaccination cards is, therefore, fundamental to reducing sources of error [17–19]. It is critical to comply with the recommended schedule to optimize protection from preventable diseases.

In addition to immunization system characteristics and the children’s background, mothers’ education was identified as impacting a child’s complete vaccination status; the reasons caregivers provided for undervaccination included parental unawareness, attitude or inability to pay vaccination fees to healthcare providers’ practices or lack of availability of the vaccine. This complexity suggests that additional efforts are needed to ensure complete vaccination for children residing in Lebanon. Adherence to the national routine vaccination schedule requires not only affordable and available vaccines but also persuasion of providers and caregivers that complete vaccination is of the utmost importance to protect a child’s health. Misperceptions seem to exist with regard to immunization and its administration indicated by many children missing their vaccination due to illness, although a mild infection should not prevent a child from receiving immunization [20].

The study results should be considered in light of potential limitations. Although the EPI methodology, which is a validated and highly recommended vaccination coverage cluster survey method, was applied, the approach was limited by the use of an old sampling frame and the lack of an existing household listing, compelling the researchers to rely on other available population estimates. Instead of using the global positioning system, fieldworkers relied on local authorities to define cluster boundaries and to choose landmarks in each area to locate study participants. Nonetheless, household surveys may be more reliable if they are well-conducted and measure vaccination coverage using different means, with each having its advantages and disadvantages [18]. In household surveys, the accuracy of the data is also dependent on the available vaccination documentation. In this study, both vaccination cards and recall of caregivers were used as sources for vaccination coverage. The lack of availability of cards and misplaced cards impeded operations; therefore, the verbal history provided by caregivers was taken into account. However, relying on recall is increasingly associated with incomplete or inaccurate vaccination documentation, and even vaccination cards may maintain low quality records of a child’s immunization status [19]. Nevertheless, the strengths of this study were the high response rate and the use of 5713 pictures of cards, which allowed better ascertainment of vaccination status despite the multiple types of vaccines used in Lebanon.

## Conclusions

The study findings suggest considerable variability in routine vaccination coverage in Lebanon. Obstacles to reaching every child in need of vaccination remain. High dropout rates suggest that many children remain susceptible to vaccine preventable diseases, particularly Syrian children. Optimizing coverage levels will require vaccination initiatives targeting both refugee children and children from vulnerable host communities, increased cooperation between public and private vaccine providers, training for vaccine providers to adhere to complete vaccine administration and increased awareness among caregivers.

## Additional file

Additional file 1: Expanded Programme of Immunization Cluster Survey 2015. Survey questionnaire. (DOCX 195 kb)

## Abbreviations

CES: Coverage evaluation survey
CI: Confidence interval
DTP: Diphtheria, tetanus, pertussis
EPI: Expanded Programme on Immunization
HepB: Hepatitis B
Hib: *Haemophilus influenzae* type b
IPV: Inactivated polio vaccine
MCV: Measles-containing vaccine
MMR: Measles, mumps, rubella
MoPH: Ministry of Public Health
OPV: Oral polio vaccine
RCV: Rubella-containing vaccine
UNICEF: United Nations Children’s Fund
WHO: World Health Organization
