# Supplementary material for: Vaccination coverage in Lebanon following the Syrian crisis: results from the district-based immunization coverage evaluation survey 2016
Source: BMC Public Health. 2019 Jan 14;19:58. doi: 10.1186/s12889-019-6418-9 (PMC6332691; doi:10.1186/s12889-019-6418-9)
Supplement: Supplementary file 1 — Expanded Programme of Immunization Cluster Survey 2015. Survey questionnaire. (DOCX 195 kb) [file 12889_2019_6418_MOESM1_ESM.docx]

**Expanded Programme of Immunization Cluster Survey 2015**

| **Governorate** |  | **District** |  | **Cadastre** | |  | | **Cluster ID** |  | **P-Code (IS/CS)** |  | **Serial Number** |
| --- | --- | --- | --- | --- | --- | --- | --- | --- | --- | --- | --- | --- |
|  |  |  |  | |  | |  |  |  |  |  |  |

|  |  | |
| --- | --- | --- |
| Interviewer  *(Name and Team Number)* |  |  |
|  |  | |
| Supervisor  *(Name and Number)* |  |  |
|  |  |  |
| Data Entry Personnel  *(Name and Number)* |  |  |
|  |  | |
| Date of Interview | Date: ______(DD)/ ______(MM)/______(YYYY) | |
| Household Final Interview Status | [__] Complete Interview [__] Partial Interview | |

**Supervisor’s Questionnaire Quality Control Check**

[__] Consent collected [__] All answers legible

[__] Child was 12 to 59 months old [__] Dates filled correctly

[__] Questionnaire complete [__] Skip patterns used properly

[__] Picture of vaccination card taken [__] Serial number shows on each page


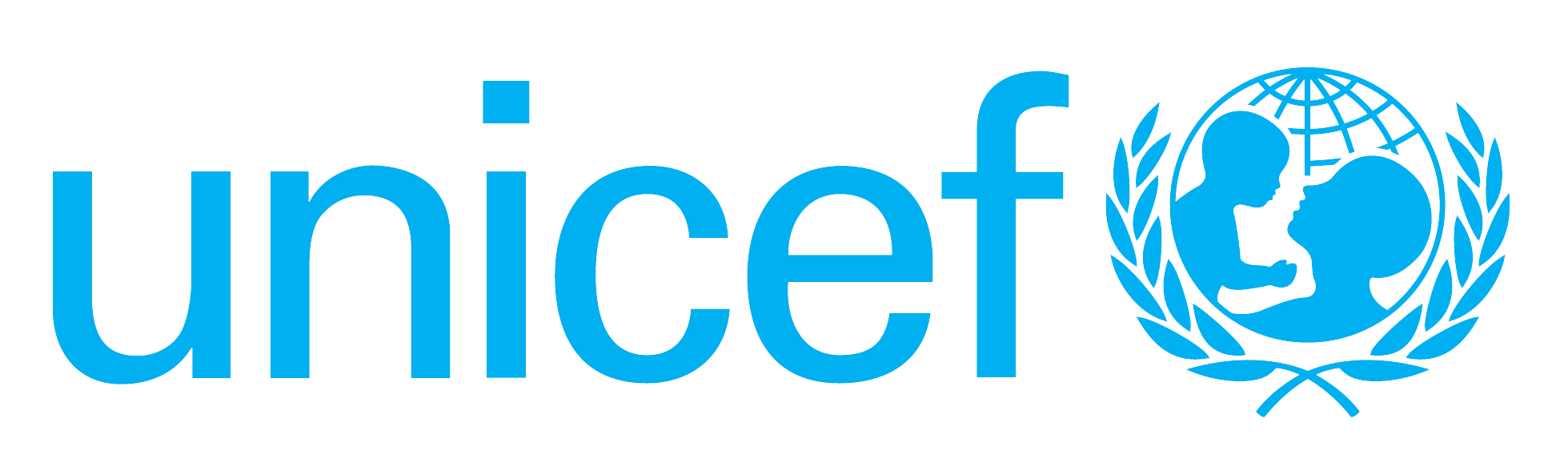

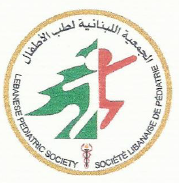

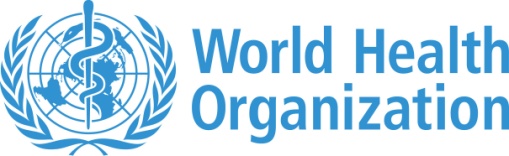

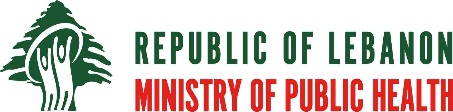
 **Signature**: ___________________ **Date:** ___________________

| **Part I: Information on the Child’s Immunization Status** |
| --- |

1. How many children between 12 months and 59 months (completed) of age live in this household? ____________ child/children
2. If there is more than one child who is between 12 months and 59 months (completed), select one by drawing a random piece of paper *(Facilitator should write the ages of all children on separate papers and draw one)*. Write the number of the selected child here. If only one child, write ‘1’: ____________
3. How are you related to the child? *(Facilitator should mention the first name/nickname of the selected child throughout the questions)*

| Legal guardian | 3 | Father | 1 |
| --- | --- | --- | --- |
| Other, please specify? ____________________ | 98 | Mother | 2 |

1. Is this the______ in the household?

| Doesn’t know/Doesn’t remember | 4 | First child | 1 |
| --- | --- | --- | --- |
| Refused to answer | 98 | Second child | 2 |
|  |  | Third or more child | 3 |

1. What is your nationality?

| Other, please specify? ___________ | 3 | Lebanese *(Move to Q7)* | 1 |
| --- | --- | --- | --- |
| Refused to answer*(Move to Q7)* | 98 | Syrian | 2 |

1. When did you come to Lebanon?

| Refused to answer | 98 | **________/ _______**  **Month Year** | 1 |
| --- | --- | --- | --- |

1. Gender of the child:

| 1 | Male | 2 | Female |
| --- | --- | --- | --- |

1. What is your child’s date of birth? ______(DD)/ ______(MM)/______(YYYY) *(If information collected, Move to Q10)*
2. If the date of birth is missing, what is the age of your child?

| Refused to answer | 98 | **________ OR _______**  **Months Years** | 1 |
| --- | --- | --- | --- |

1. What is the nationality of the child?

| Other, please specify? ___________ | 3 | Lebanese | 1 |
| --- | --- | --- | --- |
| Refused to answer | 98 | Syrian | 2 |

1. Was your child born in Lebanon?

| Doesn’t know/Doesn’t remember *(Move to Q14)* | 3 | Yes *(Move to Q14)* | 1 |
| --- | --- | --- | --- |
| Refused to answer *(Move to Q14)* | 98 | No | 2 |

1. Was your child being vaccinated in the country of birth?

| 1 | Yes | 4 | Doesn’t know/Doesn’t remember *(Move to Q14)* |
| --- | --- | --- | --- |
| 2 | No*(Move to Q14)* | 98 | Refused to answer *(Move to Q14)* |
| 3 | The child was not yet at the age of vaccination *(Move to Q14)* | | |

1. Where was your child being vaccinated most of the times (vaccines) in the country of birth?

| 1 | Outpatient clinic | 6 | Informal settlement |
| --- | --- | --- | --- |
| 2 | Private clinic | 7 | Other, can you please specify? ________________ |
| 3 | Mobile clinic | 8 | Doesn’t know/Doesn’t remember |
| 4 | Health facility | 98 | Refused to answer |
| 5 | At home |  |  |

1. Has your child ever been vaccinated?

| 1 | Yes *(Move to Q16)* | 3 | Doesn’t know/Doesn’t remember *(Move to Q16)* |
| --- | --- | --- | --- |
| 2 | No | 98 | Refused to answer*(Move to Q16)* |

1. Why your child hasn’t ever been vaccinated? *(Facilitator should code the answer at the end of the interview from the list figuring at the end of the questionnaire) (Answer and Move to Q53)*

Main reason: ________________________________________________________

Secondary reasons *(if the respondent volunteers to give other reasons)*: ___________________________________________________________________

1. Do you think that your child’s vaccination is up-to-date for his/her age?

| 1 | Yes | 3 | Doesn’t know/Doesn’t remember |
| --- | --- | --- | --- |
| 2 | No | 98 | Refused to answer |

1. Have you ever received a vaccination card or health record for your child?

| 1 | Yes, seen *(Move to Q19)* | 4 | Doesn’t know/Doesn’t remember *(Move to Q19)* |
| --- | --- | --- | --- |
| 2 | Yes, not seen *(Move to Q19)* | 98 | Refused to answer *(Move to Q19)* |
| 3 | No |  |  |

1. Why don’t you have a vaccination card or health record for your child?

| 1 | You were never given one *(Move to Q22)* | 4 | Doesn’t know/Doesn’t remember *(Move to Q22)* |
| --- | --- | --- | --- |
| 2 | Your doctor/health facility keeps it *(Move to Q20)* | 98 | Refused to answer *(Move to Q20)* |
| 3 | It is lost *(Move to Q22)* |  |  |

1. Do you take the vaccination card or health record of your child with you when you visit the doctor/health facility for vaccination?

| 1 | Always | 5 | You’re not the one taking the child for vaccination |
| --- | --- | --- | --- |
| 2 | Often | 6 | Never |
| 3 | Sometimes | 7 | Doesn’t know/Doesn’t remember |
| 4 | Rarely | 98 | Refused to answer |

1. Does the doctor or nurse register the administered vaccine on the vaccination card or health record?

| 1 | Always | 5 | Never |
| --- | --- | --- | --- |
| 2 | Often | 6 | Doesn’t know/Doesn’t remember |
| 3 | Sometimes | 98 | Refused to answer |
| 4 | Rarely |  |  |

1. Does this vaccination card have all the vaccinations that your child received recorded?

| 1 | Yes | 3 | Doesn’t know/Doesn’t remember |
| --- | --- | --- | --- |
| 2 | No | 98 | Refused to answer |

1. Where was your child vaccinated during the last visit for vaccination?

| 1 | Private hospital | 6 | At home |
| --- | --- | --- | --- |
| 2 | Public hospital | 7 | Informal settlement |
| 3 | Private clinic | 8 | Other, can you please specify? ________________ |
| 4 | Mobile clinic | 9 | Doesn’t know/Doesn’t remember |
| 5 | Health facility/PHC | 98 | Refused to answer |

1. When your visit the health clinic/ center / doctor, do you usually receive any advice on when to bring your child for next vaccination?

| 1 | Always | 5 | Never |
| --- | --- | --- | --- |
| 2 | Often | 6 | Doesn’t know/Doesn’t remember |
| 3 | Sometimes | 98 | Refused to answer |
| 4 | Rarely |  |  |

1. Has your child received any oral polio vaccine through routine vaccinations?

| Doesn’t know/Doesn’t remember *(Move to Q27)* | 3 | No *(Move to Q26)* | 2 | Yes | 1 |
| --- | --- | --- | --- | --- | --- |

1. How many times? ______________ *(Move to Q27)*
2. If no, what was the main reason? ________________________________________

Secondary reasons *(if the respondent volunteers to give other reasons)*: ___________________________________________________________________

1. Has your child received any injected polio vaccine?

| Doesn’t know/Doesn’t remember *(Move to Q30)* | 3 | No *(Move to Q29)* | 2 | Yes | 1 |
| --- | --- | --- | --- | --- | --- |

1. How many times? ______________ *(Move to Q30)*
2. If no, what was the main reason? ________________________________________

Secondary reasons *(if the respondent volunteers to give other reasons)*: ___________________________________________________________________

1. Has your child received vaccination during supplemental Polio Campaigns? (*Facilitator should mention when some of these campaigns took place to remind parents about them*)

| Doesn’t know/Doesn’t remember *(Move to Q33)* | 3 | No *(Move to Q32)* | 2 | Yes | 1 |
| --- | --- | --- | --- | --- | --- |

1. How many times? ______________ *(Move to Q33)*
2. If no, what was the main reason? ________________________________________

Secondary reasons *(if the respondent volunteers to give other reasons)*: ___________________________________________________________________

1. Has your child received any Diphtheria, Tetanus, and Pertussis (DTP)/Pentavalent vaccine?

| Doesn’t know/Doesn’t remember *(Move to Q36)* | 3 | No *(Move to Q35)* | 2 | Yes | 1 |
| --- | --- | --- | --- | --- | --- |

1. How many times? ______________ *(Move to Q36)*
2. If no, or less than 3 times, what was the main reason? ________________________

Secondary reasons *(if the respondent volunteers to give other reasons)*: ___________________________________________________________________

1. Has your child received any vaccine injected in the thigh?

| Doesn’t know/Doesn’t remember | 3 | No | 2 | Yes | 1 |
| --- | --- | --- | --- | --- | --- |

1. Has your child received any Hepatitis B vaccine?

| Doesn’t know/Doesn’t remember *(Move to Q40)* | 3 | No *(Move to Q39)* | 2 | Yes | 1 |
| --- | --- | --- | --- | --- | --- |

1. How many times? ______________ *(Move to Q40)*
2. If no, or less than 3 times, what was the main reason? ________________________

Secondary reasons *(if the respondent volunteers to give other reasons)*: ___________________________________________________________________

1. Has your child received the Hepatitis B birth dose (zero dose)?

| Doesn’t know/Doesn’t remember | 3 | No | 2 | Yes | 1 |
| --- | --- | --- | --- | --- | --- |

1. Has your child received any Haemophilus influenzae type b (Hib) vaccine?

| Doesn’t know/Doesn’t remember *(Move to Q44)* | 3 | No *(Move to Q43)* | 2 | Yes | 1 |
| --- | --- | --- | --- | --- | --- |

1. How many times? ______________ *(Move to Q44)*
2. If no, or less than 3 times, what was the main reason? ________________________

Secondary reasons *(if the respondent volunteers to give other reasons)*: ___________________________________________________________________

1. Has your child received any measles vaccine?

| Doesn’t know/Doesn’t remember | 3 | No | 2 | Yes *(Move to Q46)* | 1 |
| --- | --- | --- | --- | --- | --- |

1. If no, what was the main reason? ________________________________________

Secondary reasons *(if the respondent volunteers to give other reasons)*: ___________________________________________________________________

1. Has your child received any Measles, Mumps, and Rubella (MMR) vaccine?

| Doesn’t know/Doesn’t remember *(Move to Q49)* | 3 | No *(Move to Q48)* | 2 | Yes | 1 |
| --- | --- | --- | --- | --- | --- |

1. How many times? ______________ *(Move to Q49)*
2. If no, what was the main reason? ________________________________________

Secondary reasons *(if the respondent volunteers to give other reasons)*: ___________________________________________________________________

1. How many vaccination cards are available?

| Doesn’t know/Doesn’t remember | 4 | One card | 1 |
| --- | --- | --- | --- |
| Refused to answer | 98 | Two cards | 2 |
|  |  | Three or more cards | 3 |

1. Take a picture of the following information from the vaccination card(s)/health record(s): *(The facilitator will take a picture of the vaccination card/health record and the information will be filled within the Research Center offices)*

| If given | | | Given | |  |
| --- | --- | --- | --- | --- | --- |
| Year | Month | Day | No | Yes |  |
|  |  |  |  |  | Oral Polio 1^st^ dose |
|  |  |  |  |  | Oral Polio 2^nd^ dose |
|  |  |  |  |  | Oral Polio 3^rd^ dose |
|  |  |  |  |  | IPV 1^st^ dose |
|  |  |  |  |  | IPV 2^nd^ dose |
|  |  |  |  |  | IPV 3^rd^ dose |
|  |  |  |  |  | DTP 1^st^ dose |
|  |  |  |  |  | DTP 2^nd^ dose |
|  |  |  |  |  | DTP 3^rd^ dose |
|  |  |  |  |  | Hepatitis B 0 dose |
|  |  |  |  |  | Hepatitis B 1^st^ dose |
|  |  |  |  |  | Hepatitis B 2^nd^ dose |
|  |  |  |  |  | Hepatitis B 3^rd^ dose |
|  |  |  |  |  | Hib 1^st^ dose |
|  |  |  |  |  | Hib 2^nd^ dose |
|  |  |  |  |  | Hib 3^rd^ dose |
|  |  |  |  |  | Measles |
|  |  |  |  |  | MMR 1^st^ dose |
|  |  |  |  |  | MMR 2^nd^ dose |
|  |  |  |  |  | Booster(s)  ______________________  ______________________  ______________________  ______________________ |
| The picture(s) of the vaccination card(s)/health record was taken at:   \| Health facility  Name of the facility: _____________________________  Address of the facility: ___________________________ \| 2 \| Home \| 1 \| \| --- \| --- \| --- \| --- \| | | | | | |

1. Number of cards photographed: ____________
2. Total numbers of pictures: ____________
3. Do you know the number of times your child should be taken for vaccination to complete all the vaccines before reaching 1 year of age?

| 1 | Yes, please specify the number: _________________ | | |
| --- | --- | --- | --- |
| 2 | No |  |  |
| 98 | Refused to answer |  |  |

1. How likely do you think your child will become sick if he/she is not immunized?

| 1 | Extremely likely | 5 | Extremely unlikely |
| --- | --- | --- | --- |
| 2 | Likely | 6 | Doesn’t know/Doesn’t remember |
| 3 | Neutral | 98 | Refused to answer |
| 4 | Unlikely | | |

1. What is the main source of information you use to decide about vaccinating your child?

| 1 | Media (Radio, TV, newspaper) | 9 | Awareness session at Informal Settlements/Collective shelter |
| --- | --- | --- | --- |
| 2 | Internet and social media | 10 | Shawish in Informal Settlements |
| 3 | School | 11 | Municipality |
| 4 | Nursery | 12 | Religious centre (Church, Mosque, etc.) |
| 5 | Friend/Relative | 13 | Brochures, poster, banner, or billboard |
| 6 | Private physician | 14 | Other, can you please specify? ________________ |
| 7 | Health facility staff/Health worker | 15 | Doesn’t know/Doesn’t remember |
| 8 | Awareness session by NGO/volunteers | 98 | Refused to answer |

1. Who in the family makes decisions about vaccinating your child?

| 1 | No one | 6 | Father in law |
| --- | --- | --- | --- |
| 2 | Both parents | 7 | Other, can you please specify? ________________ |
| 3 | Mother | 8 | Doesn’t know/Doesn’t remember |
| 4 | Father | 98 | Refused to answer |
| 5 | Mother in law |  |  |

1. Did you know that in the public sector/primary healthcare centers the vaccines are given free of cost?

| Refused to answer | 98 | No | 2 | Yes | 1 |
| --- | --- | --- | --- | --- | --- |

1. Did you know that the vaccination status of your child is checked when starting school/kindergarten?

| Refused to answer | 98 | No | 2 | Yes | 1 |
| --- | --- | --- | --- | --- | --- |

| **Part II: Demographics** |
| --- |

1. What is your date of birth? ______(DD)/ ______(MM)/______(YYYY)
2. Where are you currently residing?

| 1 | Rented house/apartment | 4 | Collective shelter |
| --- | --- | --- | --- |
| 2 | Owned house/apartment | 5 | Other, please specify? ___________ |
| 3 | Informal settlement | 98 | Refused to answer |

1. What is the total number of individuals living in your household?

| 1 | ____________ individuals | 98 | Refused to answer |
| --- | --- | --- | --- |

1. What is your social status?

| 1 | Single | 4 | Widowed man |
| --- | --- | --- | --- |
| 2 | Married | 5 | Widowed woman |
| 3 | Divorced | 98 | Refused to answer |

1. What is the educational level of the child’s______?

| ***63.1*** | ***Father?*** | ***63.2*** | ***Mother?*** |
| --- | --- | --- | --- |
| 1 | Doesn’t know how to read and write | 1 | Doesn’t know how to read and write |
| 2 | Knows how to read and write | 2 | Knows how to read and write |
| 3 | Primary/complementary level | 3 | Primary/complementary level |
| 4 | Secondary level | 4 | Secondary level |
| 5 | Post school technical level | 5 | Post school technical level |
| 6 | University level | 6 | University level |
| 7 | Doesn’t know/Doesn’t remember | 7 | Doesn’t know/Doesn’t remember |
| 98 | Refused to answer | 98 | Refused to answer |

1. What is the current professional status of the______?

| ***64.1*** | ***Father?*** | ***64.2*** | ***Mother?*** |
| --- | --- | --- | --- |
| 1 | He has a full time job, please specify: _______________________ | 1 | She has a full time job, please specify: _______________________ |
| 2 | He has a part time job, please specify: _______________________ | 2 | She has a part time job, please specify: _______________________ |
| 3 | He does not work | 3 | She does not work |
| 4 | He is a retiree | 4 | She is a retiree |
| 98 | Refused to answer | 98 | Refused to answer |

1. What is your religion?

| 1 | Muslim Shiite | 4 | Christian |
| --- | --- | --- | --- |
| 2 | Muslim Sunni | 5 | Other, please specify? ___________ |
| 3 | Druze | 6 | Refused to answer |

***Observations/ Notes for Supervisor:*** ________________________________________________________________________________________________________________________________________________________________________________________________________________________________________________________________________________________________________________________________________________________________________

***Use this table to code the answers given if the child have never received any vaccine, or if any recall question was answered as “No”, or “Less than 3 times” for DTP/Pentavalent, Hepatitis B and Hib.***

| **Lack of Information** | |
| --- | --- |
| **1** | You were not aware of the need for immunization |
| **2** | You were not aware of the vaccine’s importance |
| **3** | You were not aware of the need to return for further doses |
| **4** | You did not know where to go to vaccinate your child |
| **5** | You did not know when to go to vaccinate your child |
| **6** | You were afraid of the side effects of the vaccine |
| **Lack of Motivation** | |
| **7** | You did not trust the quality of the vaccine |
| **8** | You did not trust the vaccinator (health worker) |
| **9** | You were unable to pay the fees |
| **10** | You postponed the vaccination for other times |
| **Obstacles** | |
| **11** | Your child has a chronic illness |
| **12** | Your child was sick |
| **13** | Your child’s elder siblings were sick as a result of vaccination |
| **14** | A chronically-ill person lived in your house |
| **15** | Your doctor did not advise to vaccinate |
| **16** | The school/kindergarten did not advise to vaccinate |
| **17** | The waiting time at the vaccination center was too long |
| **18** | The opening hours of vaccination centers were not convenient |
| **19** | The vaccination place was far |
| **20** | You were busy; there was nobody to bring the child to a vaccination center |
| **21** | The vaccinator was absent |
| **22** | The approach and attitude of vaccinators was repellent |
| **23** | The vaccine was not available |
| **24** | Other, please specify? ____________________________________ |
| **25** | Doesn’t know/Doesn’t remember |
| **98** | Refused to answer |
